# The relationship between physical activity, sleep duration and depressive symptoms in older adults: The English Longitudinal Study of Ageing (ELSA)

**DOI:** 10.1016/j.pmedr.2016.09.006

**Published:** 2016-09-20

**Authors:** Victoria Garfield, Clare H. Llewellyn, Meena Kumari

**Affiliations:** a Department of Epidemiology and Public Health, University College London, UK; b Institute for Social & Economic Research, University of Essex, UK

**Keywords:** Ageing, Exercise, Depression, Epidemiology, Moderation, Sleep duration

## Abstract

Research to date suggests that physical activity (PA) is associated with distinct aspects of sleep, but studies have predominantly focused on sleep quality, been carried out in younger adults, and have not accounted for many covariates. Of particular interest is also the reported relationship between physical activity and depression in older adults and as such, their associations with sleep duration. Here we examine the cross-sectional relation between physical activity and sleep duration in a community-dwelling sample of 5265 older adults from the English Longitudinal Study of Ageing. We analysed the data using multiple regression, with physical activity as a categorical exposure and sleep duration a continuous outcome, as well as testing the interaction between physical activity and depressive symptoms, which was significant (*p* < 0.001). We therefore stratified our analyses by depressive symptomatology. Our main finding was that, in the group with elevated depressive symptoms only, physical activity was positively associated with sleep duration in models adjusted for all covariates (age, sex, wealth, ethnicity, smoking, alcohol consumption, BMI, long-standing illness) across low [B (mean difference in sleep duration) = 25.22 min, 95% CI = (3.72; 46.72)], moderate [B = 27.92 min, 95% CI = (6.59; 49.26)] and high [B = 31.65 min, 95% CI = (7.36; 55.94)] PA groups, in comparison to the sedentary group. However, we observed no relation between physical activity and sleep duration in respondents who reported no depressive symptoms, irrespective of physical activity level (*p* > 0.05). Our findings suggest that a potentially effective way of improving sleep in older adults with depressive symptoms is *via* physical activity interventions.

## 1 Background

The association of physical activity (PA) with distinct aspects of sleep has been the subject of much research (Youngstedt, 2005). A study of 3081 adults from the National Health and Nutrition Examination Survey (NHANES) aged between eighteen and eighty-five years, found an inverse association between objectively-measured physical activity and: excessive daytime sleepiness, leg cramps during sleep and trouble concentrating when tired (Loprinzi and Cardinal, 2011). A Japanese population-based study of 30,000 individuals also found that a lack of exercise predicted sleep loss (Ohida et al., 2001), and a UK prospective study indicated that physical activity appears to protect against the development of insomnia in older adults (Morgan, 2003).

However, a recent meta-analysis which included open trials and RCTs advocates that physical activity has only a small beneficial effect on sleep and that this relation is moderated by age, such that it is significantly weaker in older adults (Kredlow et al., 2015). This discrepancy in results from observational data *versus* trials may relate to residual confounding in observational studies and a failure to account for potentially interesting covariates. Of particular interest is the reported association of exercise and depression in older adults, which appears to have been explored mainly in trials but less so in epidemiological studies (Mammen and Faulkner, 2013). There is also a well-established association between sleep and physical activity (Buman et al., 2011), but the interrelationships between physical activity, sleep and depression have not been properly explored. It is therefore important to investigate whether older adults might differ in their levels of physical activity dependent on whether they have depressive symptoms or not, in part due to the fact that exercise has antidepressant effects (Dunn et al., 2001), which subsequently could have a positive impact on sleep (Buman et al., 2011).

The majority of previous research in this area has not focused on physical activity and its relationship with self-reported sleep duration *per se*. Instead, these studies have placed emphasis on the association of physical activity with sleep quality (King et al., 1997) and sleep disorders (Morgan, 2003, Sherrill et al., 1998), although some have included questions about sleep duration as part of a wider sleep scale (Morgan, 2003, King et al., 1997). However, investigating sleep duration *per se* in relation to physical activity is important, as sleep duration has been associated with several adverse health outcomes (Cappuccio et al., 2010) and mortality in large-scale prospective analyses (Cappuccio et al., 2010).

Due to these shortcomings in the current literature, in this short paper we present cross-sectional findings from the English Longitudinal Study of Ageing (ELSA), in which we extend previous research, as mentioned above, by examining the association between physical activity levels and sleep duration and the role of social, psychological and physical health, as these have been associated with sleep parameters (Cappuccio et al., 2008, da Silva et al., 2016, Lallukka et al., 2012). In particular, sleep duration has been related to physical conditions, such as type 2 diabetes, hypertension, obesity, respiratory illnesses, cardiovascular disease (Cappuccio et al., 2008, da Silva et al., 2016), social factors including educational attainment and income (Michael, 2010), and psychological factors such as subjective well-being (Cappuccio et al., 2008) and self-rated health (da Silva et al., 2016).

Recent research has also found there to be a curvilinear relation between depression and sleep duration, such that both short and long sleep duration are associated with depression (van Mill et al., 2010).

To our knowledge, there has not been a study that investigates this association in a large-scale, nationally representative cohort in older adults within the UK. Our main hypotheses were i) that physical activity would be positively associated with sleep duration, such that increased physical activity is related to longer duration of sleep, and ii) that this association would potentially differ depending on whether respondents reported depressive symptoms or not.

## 2 Methods

We used data from the English Longitudinal Study of Ageing (ELSA), which is an on-going nationally representative cohort study of health and ageing initiated in 2002–3 (wave 1) (Steptoe et al., 2013). Data have been collected from participants at waves 1 (2002 − 3), 2 (2004–5), 3 (2006–7) and 4 (2008–9), 5 (2010 − 11) and 6 (2012 − 13) and comprise a representative sample of English household residents aged fifty and over. Written informed consent is obtained from participants at each wave. Questions on sleep duration and disturbance were introduced for the first time in ELSA at wave 4 and here we analyse data from 5265 respondents from wave 4, who had complete data available for all the exposure, outcome and covariates.

Physical activity and sleep duration were self-reported during the wave 4 data collection. The physical activity measure was created by summarizing the combination of responses to the level of work activity and the “type and amount of physical activity involved in daily life” questions (frequency of participation in mild/moderate/vigorous sports or activities) and categorised into: *Sedentary* (mild exercise 1–3 times a month, no moderate or vigorous activity), *Low* (mild, but no vigorous activity at least once a week), *Moderate* (moderate activity more than once a week, or vigorous activity between once a week to 1–3 times a month) and *High* (heavy manual work or vigorous activity more than once a week). Although this measure has not been assessed for reliability and validity, it has been used in previously published ELSA research (Demakakos et al., 2010, Hamer et al., 2014, Dhalwani et al., 2016).

Sleep duration was self-reported and respondents were asked to provide the “number of hours on average week night”, whilst daytime sleep duration was not assessed (Banks et al., 2010). Whilst the validity and reliability of this question has not been evaluated, self-reported sleep duration measures such as this one are commonly used in epidemiological research (Cappuccio et al., 2010, Gallicchio and Kalesan, 2009).

Self-reported demographic, socio-economic, health and health behaviour measures were used as covariates. Age was recorded as a continuous number until 90 years, with ages above 90 collapsed into the value of 91. Social and economic position was determined by quintiles of non-pension wealth, which is an important measure of social position in older age (Pollack et al., 2007). Health behaviours included frequency of alcohol consumption within the last 12 months [categorised into: less than daily; daily (5 to 7 times per week)]; and smoking status (never, ex-smoker, current smoker), whilst health measures included body mass index (BMI) (calculated from researcher-measured height and weight), self-reported long-standing illness (respondents were asked whether they had a long-standing illness, disability or infirmity, to which they responded ‘No’ or ‘Yes’), and depressive symptoms (Centre for Epidemiologic Studies – Depression Scale – CESD – 8 item scale) were also assessed. CES-D responses were summed to obtain a total score, (excluding the following item: “whether respondent felt their sleep was restless in the past week”), which was then dichotomised using a cut-point of ≥ 3 as previously described in ELSA studies (White et al., 2016).

### 2.1 Statistical analysis

All analyses were performed in STATA, version 13 and our significance threshold was set at *p* < 0.05. Analysis of variance (ANOVA) allowed us to compare means for age, sleep duration and BMI across PA categories, whilst chi-squared analyses were performed to examine differences in categorical demographic variables (sex, smoking, alcohol consumption, long-standing illness, wealth and depressive symptoms) across the 4 physical activity categories. Comparisons using ANOVA and chi-square were also made between respondents included in our sample and those who had missing data and were excluded.

First, we tested for a physical activity by age interaction, because some studies have indicated that the relationship between physical activity and sleep varies with age. Second, we tested for an interaction between physical activity and depressive symptoms. Both interactions were tested separately by means of linear regressions with sleep duration as the outcome, across 4 models with increasing adjustment for covariates: Model 1 = age, sex, wealth, ethnicity; Model 2 = Model 1 + smoking status, alcohol consumption; Model 3 = Model 1 + BMI, long-standing illness, Model 4 = Model 1 + Model 2 + Model 3. We have presented the results from fully-adjusted models for each of these interactions (physical activity*age and physical activity*depressive symptoms) in Section 3.

To investigate the cross-sectional association of physical activity with sleep duration, linear regressions were subsequently performed, in which physical activity was the exposure (independent variable) and sleep duration the outcome (dependent variable), across the same 4 models as the above. Although outliers were identified, the decision was made not to remove them, as they did not sufficiently alter the results.

## 3 Results

Those included in these analyses were wealthier and slightly older when compared to those not included (n = 5785) due to missing data (*p* < 0.001). The analytic sample was, on average, overweight, with the lowest mean BMI observed in the highest physical activity group, and respondents' durations of sleep differed significantly across the 4 categories of physical activity (*p* < 0.001). (See Table 1.)

As we found that the interaction term of physical activity by depression remained significant in a fully-adjusted model for all covariates [B (unstandardised coefficient) = − 12.54 min, 95% CI = (− 14.99; − 10.09), *p* < 0.001] we have presented the results stratified by depressive symptoms status, in Table 2. We found no evidence of an interaction between physical activity and age when adjusting for all covariates [B = 0.17 min, 95% CI = (− 0.03; 0.36), *p* = 0.09]. There was also no significant curvilinear relationship between depressive symptoms and sleep duration (*p* > 0.05).

Overall, Table 2 shows that there is no association between physical activity and sleep duration in respondents who report no depressive symptoms. Therefore, when compared to the Sedentary individuals, those in the Low, Moderate and High PA categories did not have significantly different durations of sleep (all *p* > 0.05) in any of the four models. However, the pattern of associations was markedly different for participants who reported depressive symptoms, such that there was a significant and positive relationship between PA and sleep duration throughout all 4 models (Table 2); such that for participants with elevated depressive symptoms, more PA was associated with significantly longer sleep duration. In Model 4, the most complete model, adjusted for all covariates, respondents in the Low, Moderate and High PA categories slept for significantly longer than those in the Sedentary group, [B = 25.22 min, 95% CI = (3.72; 46.72)], [B = 27.92 min, 95% CI = (6.59; 49.26)], [B = 31.65 min, 95% CI = (7.36; 55.94)], respectively.

## 4 Discussion

Findings suggest that the association of physical activity and sleep duration in older adults is only observed for participants with depressive symptoms. For this subgroup of participants, the relationship between physical activity and sleep duration was independent of health and health behaviours. Specifically, we observed that respondents who reported depressive symptoms and engaged in even low levels of physical activity, slept for twenty-five minutes longer, on average, compared to the sedentary group. Previous research in this area has not focused on physical activity and its relationship with sleep duration *per se*, but instead its association with sleep quality. However, examining physical activity in relation to sleep duration is important as a large body of evidence confirms that it is associated with disease and mortality in longitudinal studies (Cappuccio et al., 2010).

Our data show that physical activity levels are positively associated with sleep duration, and therefore support research advocating exercise as an alternative approach to sleep problems in older adults (Yang et al., 2012). This finding also accords with evidence from RCTs and open trials, which suggests that physical activity has some positive effects on sleep duration (Kredlow et al., 2015). As we observed that this relationship is moderated by depressive symptoms, future interventions to improve sleep by means of exercise ought to focus on targeting this subgroup of older adults with elevated depressive symptoms. This is in contrast to the recent meta-analytic findings that the positive impact of exercise on sleep is weak in older adults (Kredlow et al., 2015), since we should take into account whether these individuals may have depressive symptoms and therefore treat them distinctly from those who do not.

Our study possesses two important strengths: the study is well powered and rich in covariates, which allowed us to perform a comprehensive analysis of the association between physical activity and sleep duration.

Conversely, our study was not free of limitations. Both exposure and outcome measures were self-reported, as well as several covariates. We did not exclude individuals who may have been diagnosed with a sleep disorder, which could explain the skewness in our data. However, analyses were performed with and without outliers and did not affect the results. Our analysis was limited to cross-sectional data, which meant we could not examine the temporal nature of the association. Future research ought to employ longitudinal data to disentangle the potential causal relationships among physical activity, depression and sleep duration. As such, it would be possible to ascertain whether older adults reporting depressive symptoms may, over time, benefit the most from physical activity interventions to improve sleep.

These analyses serve to further understand physical activity and sleep duration in older adults. Our findings indicate that the association between physical activity levels and sleep duration in older adults differs by depression status. The reverse association with sleep duration as the exposure and physical activity as the outcome also ought to be examined, as there is some evidence for bidirectionality (Holfeld and Ruthig, 2014).

## Abbreviations

RCT: randomised control trial
NHANES: National Health and Nutrition Examination Survey
ELSA: English Longitudinal Study of Ageing
UK: United Kingdom
BMI: body mass index
PA: physical activity
CES-D: Centre for Epidemiologic Studies – Depression Scale
ANOVA: analysis of variance

## Ethics approval and informed consent

ELSA participants provided full informed written consent to take part in the study and the London Multi-centre Research Ethics Committee granted ethical approval.

## Competing interests

The authors declare no competing interests.

## Authors' contributions

VG analysed the data and wrote the manuscript. MK and CL provided comments and suggestions to the MS. All authors approved the final MS.

## Transparency document

Transparency document. Image 1

## Figures and Tables

**Table 1:** Sample characteristics of participants at wave 4 of the English Longitudinal study of Ageing.

Physical activity level					
	Sedentary	Low	Moderate	High	*P*
N = 5265	n = 163	n = 1072	n = 2830	n = 1201	
Age (years)	70.75 (8.87)	67.42 (9.80)	64.60 (8.19)	63.03 (7.48)	< 0.001

Sex					
*Male*	71 (43.56)	362 (33.77)	1298 (45.88)	630 (52.46)	< 0.001
*Female*	92 (56.44)	710 (66.23)	1531 (54.12)	571 (47.54)	
Mean sleep duration (hours)	6.47 (1.61)	6.68 (1.40)	6.92 (1.18)	6.90 (1.15)	< 0.001
BMI (kg/m^2^)	28.86 (5.48)	29.59 (5.93)	28.11 (4.89)	27.95 (4.69)	< 0.001

Smoking status					
*Never smoked*	59 (36.20)	412 (38.43)	1260 (44.52)	537 (44.71)	
*Ex-smoker*	68 (41.72)	486 (45.34)	1249 (44.13)	582 (48.46)	< 0.001
*Current smoker*	36 (22.09)	174 (16.23)	321 (11.34)	82 (6.83)	

Alcohol consumption					
*Less than daily*	139 (85.28)	884 (82.46)	2165 (76.50)	872 (72.61)	< 0.001
*Daily(5–7 times a week)*	24 (14.72)	188 (17.54)	665 (23.50)	329 (27.39)	

Long-standing illness					
*No*	94 (57.67)	778 (72.57)	2427 (85.76)	1092 (90.92)	< 0.001
*Yes*	69 (42.33)	294 (27.43)	403 (14.24)	109 (9.08)	

Depressive symptoms					
*No*	80 (49.08)	683 (63.71)	2234 (78.94)	1010 (84.10)	< 0.001
*Yes*	83 (50.92)	389 (36.29)	596 (21.06)	191 (15.90)	

Wealth quintiles					
*Lowest*	62 (38.04)	231 (21.55)	309 (10.92)	95 (7.91)	
*2*	31 (19.02)	261 (24.35)	480 (16.96)	167 (13.91)	
*3*	27 (16.56)	234 (21.83)	602 (21.27)	227 (18.90)	< 0.001
*4*	19 (11.66)	182 (16.98)	725 (25.62)	285 (23.73)	
*Highest*	24 (14.72)	164 (15.30)	714 (25.23)	427 (35.55)	

BMI = Body Mass Index; Means (SDs) or n (%).

**Table 2:** Cross-sectional associations between physical activity and sleep duration in n = 5265 ELSA participants.

		Sedentary (n = 163)	Low (n = 1072)	Moderate (n = 2830)	High (n = 1201)
*Depressive symptoms = No (n* *=* *4006)*					
		Reference (n = 80)	B (95% CI) (n = 683)	B (95% CI) (n = 2233)	B (95% CI) (n = 1010)
Sleep duration					
Model 1	Age, sex, wealth, ethnicity	0	− 5.99 (− 21.56; 9.57)	2.47 (− 12.64; 17.59)	− 4.18 (− 19.72; 11.34)
Model 2	Model 1 + smoking status, alcohol consumption	0	− 6.28 (− 21.84; 9.28)	2.88 (− 12.22; 17.98)	− 3.33 (− 18.83; 12.16)
Model 3	Model 1 + BMI long-standing illness	0	− 6.05 (− 21.61; 9.51)	2.52 (− 12.58; 17.62)	− 4.12 (− 19.63; 11.39)
Model 4	Model 1 + Model 2 + Model 3 + Model 4	0	− 5.99 (− 21.56; 9.57)	2.47 (− 12.64; 17.59)	− 4.18 (− 19.72; 11.34)


*Depressive symptoms = Yes (n* *=* *1259)*					
		Reference (n = 83)	B (95% CI) (n = 389)	B (95% CI) (n = 596)	B (95% CI) (n = 191)
Sleep duration					
Model 1	Age, sex, wealth, ethnicity	0	25.69* (4.24; 47.13)	29.45* (8.32; 50.58)	33.48* (9.51; 57.45)
Model 2	Model 1 + smoking status, alcohol consumption	0	25.67* (4.19; 47.14)	29.31* (8.14; 50.49)	33.25* (9.19; 57.30)
Model 3	Model 1 + BMI, long-standing illness	0	25.21* (3.74; 46.68)	27.10* (6.70; 49.29)	31.79* (7.58; 56.00)
Model 4	Model 1 + Model 2 + Model 3	0	25.22* (3.72; 46.72)	27.92* (6.59; 49.26)	31.65* (7.36; 55.94)

B (unstandardised coefficient) = mean difference in sleep duration (minutes) compared to Sedentary PA category (reference), 95% CI = 95% confidence interval, ^⁎^Significant contrasts for specific category *versus* reference category at *p* < 0.05.

## References

[bb0005] Banks J., Breeze E., Crawford R. (2010). Financial Circumstances, Health and Well-being of the Older Population in England [Internet]. The 2008 English Longitudinal Study of Ageing (Wave 4).

[bb0010] Buman M.P., Hekler E.B., Bliwise D.L., King A.C. (2011 Sep). Moderators and mediators of exercise-induced objective sleep improvements in midlife and older adults with sleep complaints. Health Psychol..

[bb0015] Cappuccio F.P., Taggart F.M., Kandala N.-B. (2008 May). Meta-analysis of short sleep duration and obesity in children and adults. Sleep.

[bb0020] Cappuccio F.P., D'Elia L., Strazzullo P., Miller M.A. (2010 May 1). Sleep Duration and All-cause Mortality: A Systematic Review and Meta-Analysis of Prospective Studies.

[bb0025] da Silva A.A., de Mello R.G.B., Schaan C.W., Fuchs F.D., Redline S., Fuchs S.C. (2016 Feb 17). Sleep Duration and Mortality in the Elderly: A Systematic Review with Meta-Analysis. http://bmjopen.bmj.com/lookup/doi/10.1136/bmjopen-2015-008119.

[bb0030] Demakakos P., Hamer M., Stamatakis E., Steptoe A. (2010 Sep). Low-intensity physical activity is associated with reduced risk of incident type 2 diabetes in older adults: evidence from the English Longitudinal Study of Ageing. Diabetologia.

[bb0035] Dhalwani N.N., O'Donovan G., Zaccardi F. (2016 Jan 19). Long Terms Trends of Multimorbidity and Association with Physical Activity in Older English Population. http://ijbnpa.biomedcentral.com/articles/10.1186/s12966-016-0330-9.

[bb0040] Dunn A.L., Trivedi M.H., O′Neal H.A. (2001 Jun). Physical activity dose-response effects on outcomes of depression and anxiety. Med. Sci. Sports Exerc..

[bb0045] Gallicchio L., Kalesan B. (2009 Jun). Sleep duration and mortality: a systematic review and meta-analysis. J. Sleep Res..

[bb0050] Hamer M., de Oliveira C., Demakakos P. (2014 Oct 1). Non-Exercise Physical Activity and Survival: English Longitudinal Study of Ageing. http://www.ajpmonline.org/article/S0749379714002724/fulltext.

[bb0055] Holfeld B., Ruthig J.C. (2014 Oct). A longitudinal examination of sleep quality and physical activity in older adults. J. Appl. Gerontol..

[bb0060] King A.C., Oman R.F., Brassington G.S. (1997 Jan 1). Moderate-Intensity Exercise and Self-rated Quality of Sleep in Older Adults. http://jama.jamanetwork.com/article.aspx?doi=10.1001/jama.1997.03540250040029.

[bb0065] Kredlow M.A., Capozzoli M.C., Hearon B.A., Calkins A.W., Otto M.W. (2015 Jun). The effects of physical activity on sleep: a meta-analytic review. J. Behav. Med..

[bb0070] Lallukka T., Sares-Jäske L., Kronholm E. (2012). Sociodemographic and Socioeconomic Differences in Sleep Duration and Insomnia-Related Symptoms in Finnish Adults. http://bmcpublichealth.biomedcentral.com/articles/10.1186/1471-2458-12-565.

[bb0075] Loprinzi P.D., Cardinal B.J. (2011 Dec). Association between objectively-measured physical activity and sleep, NHANES 2005–2006. Ment. Health and Phys. Act..

[bb0080] Mammen G., Faulkner G. (2013). Physical activity and the prevention of depression: a systematic review of prospective studies. Am. J. Prev. Med..

[bb0085] MA G., NP P., PR G. (2010). Who gets the best sleep? Ethnic and socioeconomic factors related to sleep complaints. Sleep Med..

[bb0090] Morgan K. (2003 Sep). Daytime Activity and Risk Factors for Late-Life Insomnia. http://doi.wiley.com/10.1046/j.1365-2869.2003.00355.x.

[bb0095] Ohida T., Kamal A.M., Uchiyama M. (2001 May 1). The influence of lifestyle and health status factors on sleep loss among the Japanese general population. Sleep.

[bb0100] Pollack C.E., Chideya S., Cubbin C., Williams B., Dekker M., Braveman P. (2007 Sep). Should health studies measure wealth? A systematic review. Am. J. Prev. Med..

[bb0105] Sherrill D.L., Kotchou K., Quan S.F. (1998 Sep 28). Association of Physical Activity and Human Sleep Disorders. http://archinte.jamanetwork.com/article.aspx?doi=10.1001/archinte.158.17.1894.

[bb0110] Steptoe A., Breeze E., Banks J., Nazroo J. (2013 Dec 1). Cohort profile: the English longitudinal study of ageing. Int. J. Epidemiol..

[bb0115] van Mill J.G., Hoogendijk W.J.G., Vogelzangs N., van Dyck R., Penninx B.W.J.H. (2010 Mar). Insomnia and sleep duration in a large cohort of patients with major depressive disorder and anxiety disorders. J. Clin. Psychiatry.

[bb0120] White J., Zaninotto P., Walters K. (2016 Apr). Duration of depressive symptoms and mortality risk: the English Longitudinal Study of Ageing (ELSA). Br. J. Psychiatry.

[bb0125] Yang P.-Y., Ho K.-H., Chen H.-C., Chien M.-Y. (2012 Sep). Exercise training improves sleep quality in middle-aged and older adults with sleep problems: a systematic review. J. Physiother..

[bb0130] Youngstedt S.D. (2005 Apr). Effects of exercise on sleep. Clin. Sports Med..

